# Structural basis underlying the autoinhibition of the formin FHOD1 and its phosphorylation-dependent activation

**DOI:** 10.1016/j.jbc.2025.111109

**Published:** 2025-12-23

**Authors:** Mokhamad Fahmi Rizki Syaban, Shafiyyah Maratush Shalihah, Yohko Kage, Hikmawan Wahyu Sulistomo, Ryu Takeya

**Affiliations:** 1 Department of Pharmacology, Faculty of Medicine, University of Miyazaki, Miyazaki, Japan; 2 Department of Pharmacology, Faculty of Medicine, Universitas Brawijaya, Malang, Indonesia

**Keywords:** actin, computer modeling, FHOD, formin, phosphorylation, protein structure, Rho (Rho GTPase), stress fiber

## Abstract

FHOD1 is a member of the formin protein family that plays a role in actin polymerization, thereby inducing stress fiber formation *in vivo*. FHOD1, like other members of the formin family, harbors the diaphanous autoregulatory domain at the C-terminal region, which engages in autoinhibitory interactions with the N-terminal diaphanous inhibitory domain. However, unlike other formins that are activated by the binding of Rho GTPases, autoinhibition of FHOD1 is released by phosphorylation at the diaphanous autoregulatory domain. The specific mechanisms underlying phosphorylation-dependent activation of FHOD1 remain to be elucidated, as the structure of the complex of the N- and C-terminal regions of FHOD1 remains unresolved. In this study, an *in silico* structural model of the autoinhibitory interaction of FHOD1 was developed using AlphaFold3. The predicted model indicated that an extended polybasic region, which is unique to the FHOD subfamily, stabilizes autoinhibitory interactions. This prediction was validated through an experimental analysis using site-directed mutagenesis. Furthermore, the extended region was implicated in the process of autoinhibition release, as expected from the findings of our previous experiments, which was successfully reinforced by the structural predictions of the phosphorylated model. These findings provide a structural basis for a unique autoinhibitory mode and the activation process of FHOD1 among formin family proteins and, at the same time, underscore the powerful utility of protein structure prediction for the refinement of our understanding of protein structures and their functional implications.

Formin is a key regulator of actin filaments, orchestrating their dynamics through the nucleation and elongation processes (1, 2, 3). Formins are characterized by the presence of formin homology 1 and 2 (FH1 and FH2) domains, which are essential for the formin-mediated nucleation and elongation of actin filaments. The FH2 domain binds directly to the actin nuclei or the plus end of an actin filament and allows or accelerates the addition of actin monomers to the ends of the actin filaments (4, 5). The FH1 domain binds to profilins and delivers actin monomers to the FH2 domain. These coordinated actions accelerate the formation and elongation of linear unbranched actin filaments comprising stress fibers, filopodia, and cytokinetic contractile rings.

Fifteen members of the formin family have been identified in mammals (1). Among these, the diaphanous (DIAPH) formin subfamily is the most well-characterized formin family. Active DIAPH1 induces the formation of actin stress fibers *in vivo*, which is achieved using the catalytic unit FH1–FH2. However, the activity of DIAPH1 is normally inhibited by the intramolecular autoinhibitory interaction between the C-terminal diaphanous autoregulatory domain (DAD) and the N-terminal FH3 domain, also known as the diaphanous inhibitory domain (DID) (6, 7). DIAPH1 activation involves several steps. In the initial phase, GTP-bound Rho binds to the GTPase-binding domain (GBD) at the N terminus of DIAPH1 (8). Subsequent conformational changes cause Rho to form additional contacts with FH3. This induces electrostatic repulsion between negative charges on DAD and Rho residues, resulting in the release of DAD from the regulatory region (9, 10). Rho binding also induces restructuring of the GBD, which precludes the simultaneous binding of DAD to the N terminus (11). Thus, the activation process of DIAPH1 has been elucidated through extensive structural studies (12).

Formin homology domain–containing protein (FHOD) subfamily constitutes a distinct subclass of the formin family that regulates actin filament polymerization (13). Although FHOD in its active form also induces stress fiber formation *in vivo*, the process of activation seems to be different from that of other formins. Unlike DIAPH1, which is activated by direct interaction with Rho GTPase, FHOD1 activation primarily occurs through the phosphorylation of specific residues in its polybasic regions by Rho-associated kinase (ROCK), which disrupts FHOD1 autoinhibitory interactions, thereby promoting actin filament elongation (14). However, the structural basis of the FHOD1 activation remains unclear. This is because although the crystal structure of FH3 itself has already been solved (15), the complex structure of FH3 and DAD has not yet been solved.

In recent years, artificial intelligence–based structure prediction tools such as AlphaFold have emerged as powerful approaches that offer rapid and accurate protein structure predictions (16, 17). Although the definitive significance of primary techniques, including X-ray crystallography, NMR spectroscopy, and cryo-electron microscopy, to determine protein structure is indisputable, the integration of artificial intelligence–based structure prediction may allow us to expand our understanding of biological processes and their underlying mechanisms (18).

In the present study, we employed AlphaFold3 (19) to elucidate the structural basis of autoinhibition of FHOD1 and its phosphorylation-dependent activation. AlphaFold3 predicted the autoinhibitory complex of the N terminus with DAD with a high degree of confidence. The predicted model was successfully validated by experimental verification, thereby providing high-resolution structural information. Furthermore, the predicted model with phosphorylated residues involved in activation enabled speculation regarding the molecular mechanisms of biological processes. These results illustrate the potential of computational predictions and experimental methods to integrate and collectively enhance our understanding of protein structures and their functional implications.

## Results

### Prediction of a structural model of the autoinhibitory complex of FHOD1

**Figure 1:**
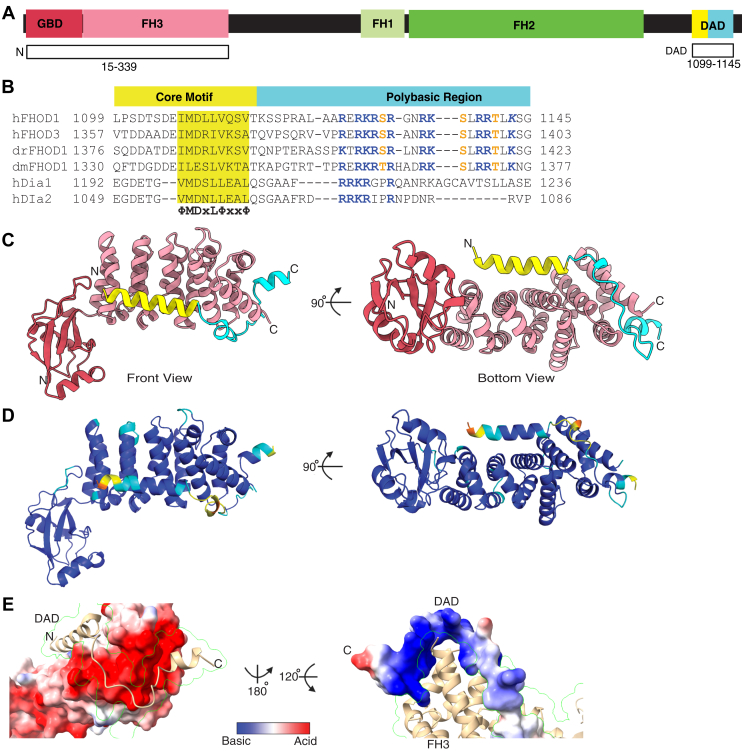
**Prediction of a structural model of the autoinhibitory complex of FHOD1.***A,* schematic representation of the domain architecture of human FHOD1. The fragments used for model prediction are indicated by *white boxes*. *B,* sequence alignment of DAD of FHOD and DIAPH proteins: dm, *Drosophila melanogaster*; dr, *Danio rerio*; and h, *Homo sapiens*. The DAD core motif (ΦMDxLΦxxΦ) is shaded in *yellow*. Basic residues and phosphorylatable Ser/Thr residues are shown in *blue* and *orange*, respectively. *C,* the predicted model of the complex structure of the N terminus and DAD of FHOD1 by AlphaFold3. *D,* the model with colors of pLDDT values. Very high (pLDDT >90), high (90 > pLDDT > 70), low (70 > pLDDT > 50), and very low (pLDDT <50) are indicated by *blue*, *cyan*, *yellow*, and *orange*, respectively. *E,* electrostatic surface representation of the predicted complex between the N terminus and DAD. *Left*, view from the DAD side with reduced opacity for the DAD domain, highlighting the negative charge on the binding surface of the N terminus. The *green lines* represent the surface contour of the DAD domain. *Right*, view from the N terminus side with reduced opacity for the N terminus, highlighting the positive charge on the binding surface of the extended polybasic region. The *green lines* represent the surface contour of the N terminus. DAD, diaphanous autoregulatory domain; DIAPH, diaphanous; FHOD, formin homology domain–containing protein; pLDDT, predicted local distance difference test.

We previously showed that the N-terminal region (1–569) containing the GBD and FH3 domains directly binds to the C-terminal DAD (1081–1145) of FHOD1 (20). Subsequently, the GBD and FH3 domains were reported to be sufficient for binding (21). In the present study, we generated a series of truncated mutants and confirmed that 1 to 360 of FHOD1 was sufficient for binding to DAD (Fig. S1, *A* and *B*). We then employed AlphaFold3 to predict the structure of the complex comprising the N-terminal region (1–360) and DAD (1081–1145). As shown in Fig. S1*C*, the initial model contains unstructured regions. Therefore, we trimmed these unstructured regions and remodeled the complex structure using the N-terminal region (15–339) and DAD (1099–1145) (Fig. 1*A*). This set of fragments was used for model prediction in subsequent analyses.

### Bipartite structure of FHOD1–DAD consisting of a core motif and a unique extended polybasic region

The predicted complex structure of the N-terminal region (15–339) and DAD (1099–1145) of FHOD1 showed that the interaction between FH3 and DAD is mediated by two separate motifs within the DAD: the core motif (ΦMDxLΦxxΦ, where Φ is a hydrophobic residue) and the basic C-terminal polybasic region (Fig. 1, *B* and *C*). The core motif harboring a hydrophobic consensus sequence formed an amphipathic α-helix and contacted the concave surface of FH3, whereas the polybasic region was bound to wrap around the C-terminal surface of FH3. The predicted model exhibited a high degree of confidence, as evidenced by its predicted local distance difference test (pLDDT) score (Fig. 1*D*), and a very low score for the predicted aligned error (Fig. S2) across all regions, including the core motif and the polybasic region, suggesting that these regions contribute structurally to the stabilization of the complex. The analysis of the predicted model using the web analysis tool PDBsum (22) confirmed the existence of two distinct interaction modes: a hydrophobic interaction mediated by the core motif and an electrostatic interaction mediated by the basic region (Fig. S3).

The basic residues following the C terminus of the core motif were also found in members of the DIAPH subfamily (Fig. 1*B*). Although this basic RRKR motif is critical for the autoinhibitory interaction of DIAPH (23, 24), the basic residues were not visible in the crystal structure of the complex between FH3 and DAD (Fig. S4*A*) (9). A recent NMR analysis has also revealed that the basic region is unstructured in the solution structure and that the binding of the basic region to FH3 is only transient (Fig. S4*B*) (25). Consistently, in the AlphaFold3-predicted structure, these basic residues of DIAPH1 were predicted with very low confidence (Fig. S4, *C* and *D*), despite their high conservation (Fig. S4*E*), which supports the unstructured conformation of the polybasic region of DIAPH1.

Unlike the DIAPH subfamily, the FHOD subfamily contains a C-terminal extension of the basic region (Fig. 1*B*). In the current structural model, this extended polybasic region of FHOD1 appeared to contribute to the binding of DAD to FH3 (Fig. 1*C*). As shown in Figure 1*E*, the complementary charge between the negatively charged FH3 and the positively charged extended region seemed to contribute to stabilizing the complex. The present predicted model is consistent with the results of previous NMR spectrometric analyses, which demonstrated that the extended region makes contact with FH3 (21). The interacting residues proposed by the NMR study were in good agreement with the interface residues predicted by the current model (Fig. S3). Thus, the FHOD subfamily–specific extended region appears to expand the electrostatically interacting surfaces to stabilize the structure of the autoinhibitory complex between FH3 and DAD.

### Importance of basic residues in the extended polybasic region of FHOD1 for autoinhibition

**Figure 2:**
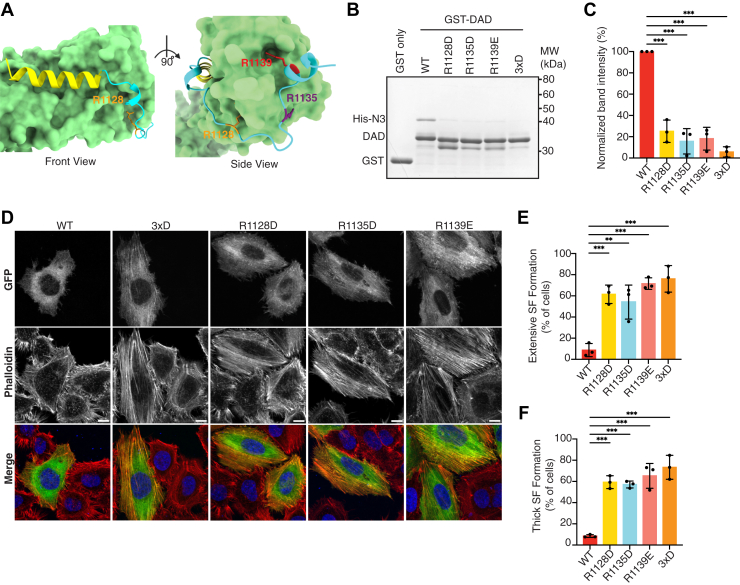
**Basic residues in the extended polybasic region of FHOD1 are essential for the autoinhibitory interactions.***A,* structures of the interfaces of the N terminus with DAD core (*left*) and with the extended polybasic region (*right*). *B,* effect of amino acid substitutions on the interaction of FHOD1–DAD with the N-terminal region. His-tagged FHOD1-N3 (1–360) was incubated with GST-fused FHOD1–DAD (1081–1145) carrying the indicated amino acid substitution: 3×D, the S1131D/S1137D/T1141D substitution. Proteins were pulled down with glutathione-Sepharose-4B, and the precipitants were subjected to SDS-PAGE, followed by CBB staining. *C,* quantification of the band intensities of FHOD1-N pulled down with DAD mutants relative to that with DAD-WT from three independent pull-down experiments. Values are means ± SD. ∗*p* < 0.05; ∗∗*p* < 0.01; and ∗∗∗*p* < 0.001. *D,* effect of amino acid substitutions on stress fiber formation. HeLa cells were transfected with a plasmid encoding GFP-FHOD1-FL carrying the indicated substitution. Cells were fixed, followed by visualization by GFP fluorescence (*green*) or phalloidin staining (*red*). The scale bars represent 10 μm. *E* and *F,* quantitative analysis of the stress fiber formation. Cells showing stress fiber formation exceeding 50% of the cell area (*E*) and cells showing more than five thick stress fibers (*F*) were counted. For each mutant, the percentages of positive cells were calculated from a total of 20 to 30 cells in a single transfection experiment, and the mean ± SD was obtained from three independent transfections. ∗*p* < 0.05; ∗∗*p* < 0.01; and ∗∗∗*p* < 0.001. CBB, Coomassie Brilliant Blue; DAD, diaphanous autoregulatory domain; FHOD, formin homology domain–containing protein; FL, full length; GST, glutathione-*S*-transferase.

Among the basic residues in the polybasic region of FHOD1, R1128, R1135, and R1139 were oriented toward FH3 and engaged in direct interaction with FH3 (Fig. 2*A*). These three Arg residues are well conserved among the FHOD subfamily (Figs. 1*B* and S5*A*), and all atoms of these residues were estimated to be very high (pLDDT score >90) or confident (pLDDT score >70) (Fig. S5*B*). The analysis of the predicted model using the Amino Acid Interactions (INTAA) web server (26) showed that these three residues contributed significantly to the stability of the protein complex (Fig. S5*C*), suggesting their critical role in stabilizing the autoinhibitory conformation. Therefore, we substituted these three basic residues with Asp or Glu and performed an *in vitro* binding assay using a glutathione-*S*-transferase (GST) pull-down assay (Fig. 2, *B* and *C*). The single substitution greatly attenuated the autoinhibitory interaction to a level comparable to that of the triple substitution of aspartate for S1131, S1137, and T1131 (3×D), which mimics the phosphorylated form (14).

We next examined stress fiber formation by overexpressing mutated full-length FHOD1 (FHOD1-FL) in HeLa cells to determine whether these mutations can activate FHOD1 *in vivo*. The ectopic expression of FHOD1-FL did not induce the formation of actin stress fibers, whereas FHOD1-FL carrying 3×D mutations led to the formation of actin stress fibers (Fig. 2*D*), consistent with our previous observation (14). Expression of FHOD1-FL carrying the substitution for the basic residues R1128, R1135, or R1139 induced the formation of actin stress fibers (Fig. 2, *D*–*F*), indicating that these single mutations for basic residues can disrupt autoinhibitory interactions, thereby activating FHOD1 *in vivo*.

### Mediation of autoinhibitory interaction with DAD by conserved acidic residues in FH3

**Figure 3:**
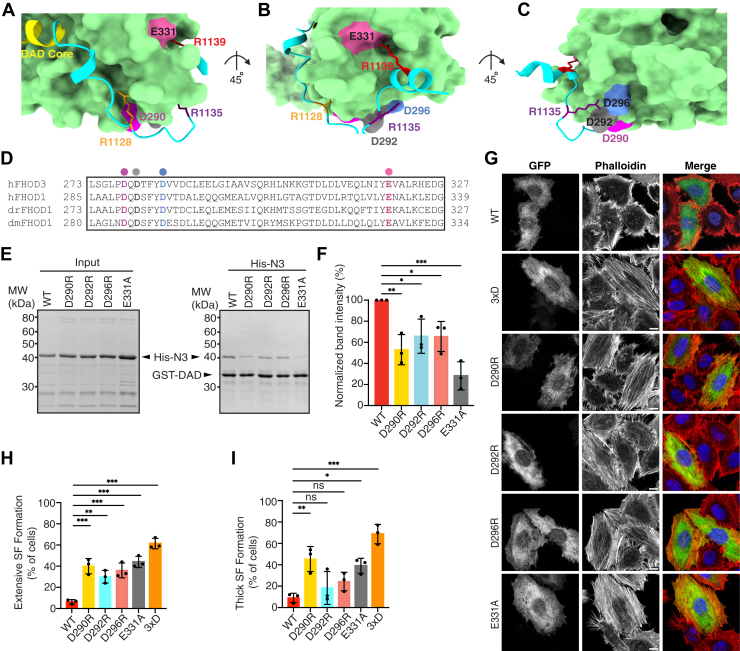
**Conserved acidic residues in the N-terminal region mediate autoinhibitory interactions of FHOD1.***A*–*C,* structures of the interfaces between the N terminus and DAD. *D,* sequence alignment of the area in the N terminus of FHOD that contacts the extended polybasic region: dm, *Drosophila melanogaster*; dr, *Danio rerio*; and h, *Homo sapiens*. Conserved acidic residues D290, D292, D296, and E331 are shown in the same color as (*A*–*C*). *E,* effect of amino acid substitutions on the interaction of FHOD1–DAD with the N-terminal region. His-tagged FHOD1-N3 (1–360) proteins carrying the indicated amino acid substitution (*left panel*) were incubated with GST-fused FHOD1–DAD (1081–1145). Proteins were pulled down with glutathione-Sepharose-4B, and the precipitants were subjected to SDS-PAGE, followed by CBB staining (*right panel*). *F,* quantification of the band intensities of FHOD1-N mutants pulled down with DAD relative to that of FHOD1-N-WT from three independent pull-down experiments. Values are means ± SD. ∗*p* < 0.05; ∗∗*p* < 0.01; and ∗∗∗*p* < 0.001. *G,* effect of amino acid substitutions on stress fiber formation. HeLa cells were transfected with a plasmid encoding GFP-FHOD1-FL carrying the indicated substitution. Cells were fixed followed by visualization by GFP fluorescence (*green*) or phalloidin staining (*red*). The scale bars represent 10 μm. *H* and *I,* quantitative analysis of the stress fiber formation. Cells showing stress fiber formation exceeding 50% of the cell area (*H*) and cells showing more than five thick stress fibers (*I*) were counted. For each mutant, the percentages of positive cells were calculated from a total of 20 to 30 cells in a single transfection experiment, and the mean ± SD was obtained from three independent transfections. ∗*p* < 0.05; ∗∗*p* < 0.01; ∗∗∗*p* < 0.001; and ns, not significant. CBB, Coomassie Brilliant Blue; DAD, diaphanous autoregulatory domain; FHOD, formin homology domain–containing protein; FL, full length; GST, glutathione-*S*-transferase.

To confirm the contribution of R1128, R1135, and R1139 to the autoinhibitory interaction, we introduced mutations in the contacting surface of the FH3 domain. Based on the predicted model and structural analysis using PDBsum (Fig. S3*C*), we selected acidic residues D290, D292, D296, and E331, which are not buried in the helix but rather exposed on the surface and contact R1128, R1135, and R1139 in the structural model (Fig. 3, *A*–*C*). These four acidic residues were well conserved among the FHOD subfamily (Fig. 3*D*). Single substitution of these acidic residues considerably decreased the autoinhibitory interaction (Fig. 3, *E* and *F*). Expression of FHOD1-FL carrying a single substitution for these acidic residues also induced the formation of actin stress fibers (Fig. 3, *G*–*I*). The effects of acidic residue substitutions within the FH3 domain were weaker than those of basic residue substitutions in the extended polybasic region. This is likely because, as shown in Fig. S3*C*, basic residues R1128, R1135, and R1139 interact with two or three acidic residues in the FH3 domain, respectively. The results of these substitution experiments highlight the essential role of electrostatic interactions between the extended polybasic region of DAD and the negatively charged surface of the FH3 domain of FHOD1. These experimental results validated the confidence in the predicted structural model.

### Role of hydrophobic residues in the DAD core motif of FHOD1 for autoinhibitory interactions

**Figure 4:**
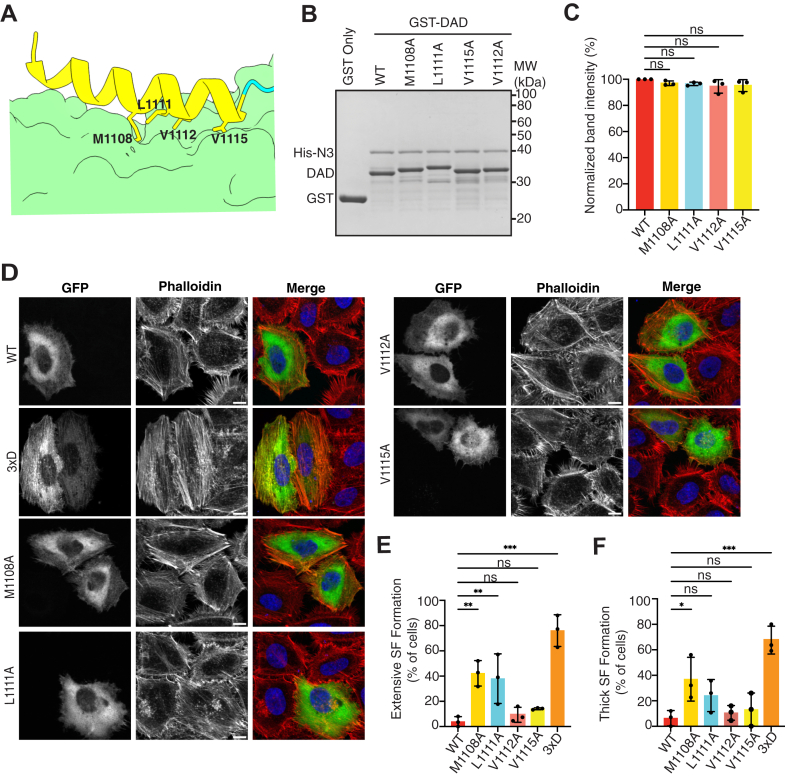
**Effect of substitutions for hydrophobic residues in the DAD core motif of FHOD1.***A,* structures of the interfaces between the DAD core motif and the N terminus of FHOD1. *B,* the effect of amino acid substitutions on the interaction of FHOD1–DAD with the N-terminal region. His-tagged FHOD1-N3 (1–360) was incubated with GST-fused mutant FHOD1–DAD (1081–1145) carrying the indicated amino acid substitution. Proteins were pulled down with glutathione-Sepharose-4B, and the precipitants were subjected to SDS-PAGE, followed by CBB staining. *C,* quantification of the band intensities of FHOD1-N pulled down with DAD mutants relative to that with DAD-WT from three independent pull-down experiments. Values are means ± SD. *D,* effect of amino acid substitutions on stress fiber formation. HeLa cells were transfected with a plasmid encoding GFP-FHOD1-FL carrying the indicated substitution. Cells were fixed followed by visualization by GFP fluorescence (*green*) or phalloidin staining (*red*). The scale bars represent 10 μm. *E* and *F,* quantitative analysis of the stress fiber formation. Cells showing stress fiber formation exceeding 50% of the cell area (*E*) and cells showing more than five thick stress fibers (*F*) were counted. For each mutant, the percentages of positive cells were calculated from a total of 20 to 30 cells in a single transfection experiment, and the mean ± SD was obtained from three independent transfections. ∗*p* < 0.05; ∗∗*p* < 0.01; ∗∗∗*p* < 0.001; and ns, not significant. CBB, Coomassie Brilliant Blue; DAD, diaphanous autoregulatory domain; FHOD, formin homology domain–containing protein; FL, full length; GST, glutathione-*S*-transferase.

Given the significant contribution of the extended polybasic region to the autoinhibitory interaction of FHOD1, we explored the role of the DAD core motif in the autoinhibitory interaction of FHOD1. In the crystal and solution structures of the complexes between DAD and FH3 of DIAPH1, the DAD core motif formed an amphipathic α-helix and contacted the concave hydrophobic surface (9, 25) (Fig. S4, *A* and *B*). The substitution of Met with Ala in the core motif of DIAPH1 resulted in a significant decrease in affinity (9), thereby abrogating the autoinhibitory interaction to induce stress fiber formation (7). In the case of FHOD1, the complex structure remains unsolved; however, previous NMR spectroscopic analyses indicated a helical conformation for the core motif (21). Consistent with this, in the current model, the core motif of FHOD1 formed an amphipathic α-helix that contacted the concave hydrophobic surface (Fig. 4*A*).

We introduced point mutations in hydrophobic residues, M1108, L1111, V1112, and V1115, which are located on the interaction surface. Unlike in the case of DIAPH1, single substitutions in the core motif did not affect interaction with FH3 in the pull-down assay, at least at a single concentration (Fig. 4, *B* and *C*). In an attempt to detect minor differences, we compared the FH3 binding of DAD-WT and DAD-M1108A at multiple protein concentrations (Fig. S6). However, no significant differences were observed, although the M1108A mutant showed a slight tendency toward low affinity. In line with the pull-down assay, the stress fiber assay showed that FHOD1-FL carrying these substitutions in hydrophobic residues failed to induce stress fiber formation as induced by the active 3×D mutant, although M1108A could induce partial activation (Fig. 4, *D*–*F*). This partial activation by M1108A appears to be consistent with the previous report that V228 is involved in the DAD–FH3 interaction in FHOD1 (15), since M1108 is predicted to interact with V228 in our predicted model (Fig. S3*B*), further supporting the reliability of the predicted structural model. Nevertheless, the contribution of the DAD core motif in the DAD–FH3 interaction appears to be less substantial than that of the extended polybasic region. These findings suggest that the contribution of the core motif to the autoinhibitory interaction differs between DIAPH and FHOD, highlighting the importance of the extended polybasic region of FHOD1 in the regulation of autoinhibition.

### Effect of phosphorylation of serine and threonine residues within the DAD on the autoinhibitory interaction

**Figure 5:**
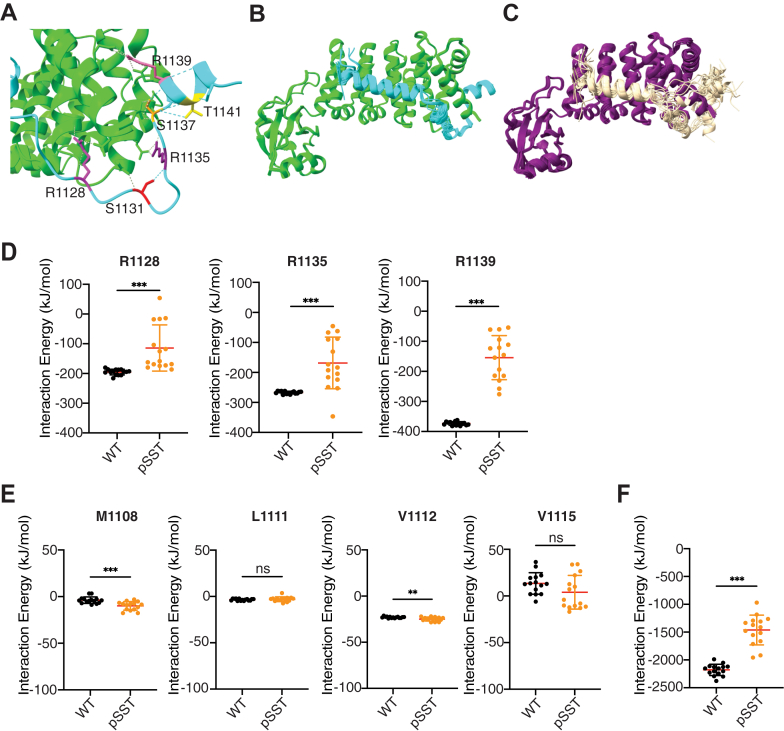
**Effect of phosphorylation at S1131, S1137, and T1141 within the extended polybasic region of FHOD1.***A,* structures of the interfaces between the extended polybasic region and the N terminus of FHOD1. The relative positions of the basic residues R1128, R1135, R1139, and the phosphorylatable residues S1131, S1137, T1141 are indicated. *B* and *C,* an overlay of 15 predicted models of the complex of unmodified DAD (*B*) and those of phosphorylated DAD at S1131, S1137, and T1141 (*C*). *D* and *E,* residue-specific interaction energy in the complex of the N terminus and DAD. The interaction energies of the basic residues R1128, R1135, and R1139 in the extended polybasic region (*D*) and the hydrophobic residues M1108, L1111, V1112, and V1115 (*E*) are calculated by the INTAA web server using 15 predicted models for unmodified DAD (WT) and DAD phosphorylated at S1131, S1137, and T1141 (pSST), respectively. Data are presented as dot plots and mean ± SD. ∗*p* < 0.05; ∗∗*p* < 0.01; ∗∗∗*p* < 0.001; and ns, not significant. *F,* the sum of interaction energies between the residues in the N terminus and DAD was calculated by the INTAA web server using 15 predicted models for unmodified DAD (WT) and DAD phosphorylated at S1131, S1137, and T1141 (pSST), respectively. FHOD, formin homology domain–containing protein; INTAA, Amino Acid Interactions.

It has been demonstrated that ROCK-mediated phosphorylation at S1131, S1137, and T1141 within the extended polybasic region of FHOD1 is sufficient to release the autoinhibitory interaction and activate FHOD1 (14). However, the structural mechanism by which phosphorylation induces the release of the autoinhibitory interaction remains unknown. In the present predicted model, these three S/T residues were in close proximity to R1128, R1135, and R1139, the critical residues for the autoinhibitory interaction (Fig. 5*A*), indicating the possibility that phosphorylation of these residues directly affects the autoinhibitory interaction.

To obtain structural information regarding the effect of phosphorylation, we introduced phosphorylation at S1131, S1137, and T1141 of the query sequence of DAD, performed structural predictions, and compared them with the unmodified structure. The overlaying of the 15 predicted models of the unmodified structure exhibited an almost overlapping structure of the entire length of the DAD (Fig. 5*B*). In contrast, the phosphorylated structure exhibited noticeable conformational diversities among the 15 models, particularly in the extended polybasic region with very low confidence (Figs. 5*C* and S7), suggesting increased flexibility in this region.

To ascertain the effect of phosphorylation at the extended region on complex destabilization, we calculated the interaction energies of each amino acid located on the interaction surface using INTAA, for both models: unmodified DAD and DAD phosphorylated at S1131, S1137, and T1141. The interaction energies of the critical basic residues R1128, R1135, and R1139 were significantly decreased by phosphorylation (Fig. 5*D*), whereas those of hydrophobic residues in the DAD core motif did not exhibit a significant decrease in energy upon phosphorylation; rather, the energies of some residues were increased slightly but significantly (Fig. 5*E*). The sum of interaction energies between the residues in DAD and the N terminus, calculated by INTAA, was also significantly reduced in the phosphorylated models (Fig. 5*F*). It is important to note that these values are not equivalent to the thermodynamic binding free energy because of the nature of the calculation (26). However, a significant decrease by phosphorylation was also observed in the predicted binding energy calculated using PRODIGY, a web server specializing in predicting the binding strength of protein–protein complexes (27) (Fig. S8), supporting destabilization of autoinhibitory binding. The predicted binding free energy (Δ*G*) of −10.7 kcal/mol for WT by PRODIGY was reasonably similar to the previously reported experimental value of −8.0 kcal/mol (21). Thus, it is suggested that phosphorylation primarily affects the electrostatic interaction mediated by the polybasic region, and the phosphorylation-mediated disruption of electrostatic interactions appears to be sufficient to release autoinhibition.

### Involvement of GBD in the autoinhibitory interaction of FHOD1

**Figure 6:**
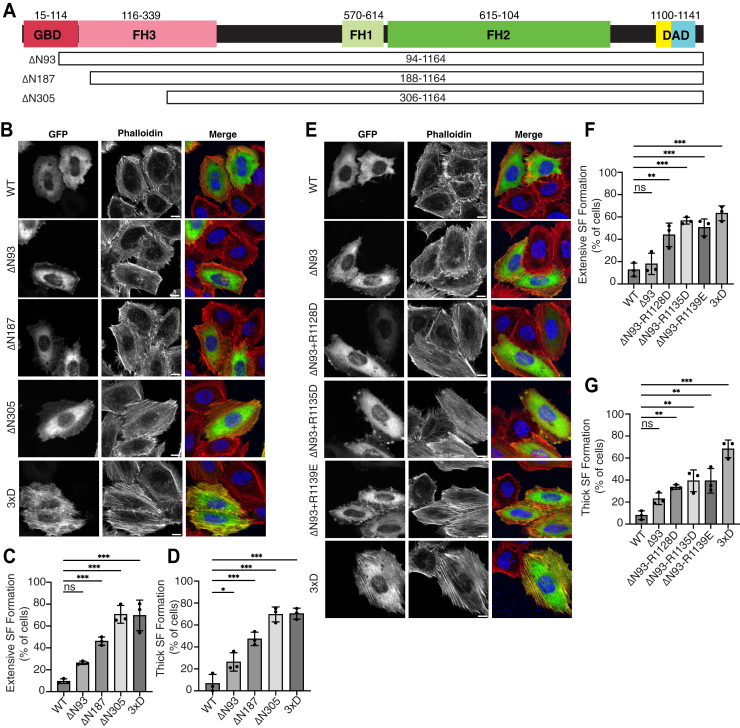
**Role of the GBD of FHOD1 in the regulation of stress fiber formation.***A,* schematic representation of the domain architecture of human FHOD1. The fragments used are indicated by *white boxes*. *B,* effect of the N-terminal deletion on stress fiber formation. HeLa cells were transfected with a plasmid encoding the indicated fragments. Cells were fixed, followed by visualization by GFP fluorescence (*green*) or phalloidin staining (*red*). The scale bars represent 10 μm. *C* and *D,* quantitative analysis of the stress fiber formation. Cells showing stress fiber formation exceeding 50% of the cell area (*C*) and cells showing more than five thick stress fibers (*D*) were counted. For each mutant, the percentages of positive cells were calculated from a total of 20 to 30 cells in a single transfection experiment, and the mean ± SD was obtained from three independent transfections. ∗*p* < 0.05; ∗∗*p* < 0.01; ∗∗∗*p* < 0.001; and ns, not significant. *E,* effect of a single substitution of critical residues R1128, R1135, and R1139 into the ΔN93 mutant on stress fiber formation. HeLa cells were transfected with a plasmid encoding the indicated mutants. Cells were fixed, followed by visualization by GFP fluorescence (*green*) or phalloidin staining (*red*). The scale bars represent 10 μm. *F* and *G,* quantitative analysis of the stress fiber formation. Cells showing stress fiber formation exceeding 50% of the cell area (*F*) and cells showing more than five thick stress fibers (*G*) were counted. For each mutant, the percentages of positive cells were calculated from a total of 20 to 30 cells in a single transfection experiment, and the mean ± SD was obtained from three independent transfections. ∗*p* < 0.05; ∗∗*p* < 0.01; ∗∗∗*p* < 0.001; and ns, not significant. FHOD, formin homology domain–containing protein; GBD, GTPase-binding domain.

Finally, we investigated whether the GBD of FHOD1 is involved in autoinhibitory interactions. In the case of DIAPH, the autoinhibitory interaction between the N terminus and DAD was released by the binding of RhoA to GBD (9). However, the GBD of FHOD1 did not appear to contribute to DAD binding (15, 28). The present predicted complex structure of FHOD1 also showed that the interaction with DAD is mediated only by FH3 within the N terminus of FHOD1. To examine whether GBD is required for FHOD1 autoinhibition, we generated a series of GBD-deleted constructs (Fig. 6*A*) and examined their activity in cells using a stress fiber assay. Deletion of the N-terminal 187 or 305 amino acids induced stress fiber formation, whereas deletion of 93 residues was not sufficient to activate FHOD1 (Fig. 6, *B*–*D*). The additional single substitution of critical residues R1128, R1135, or R1139 into the ΔN93 mutant led to robust stress fiber formation (Fig. 6, *E* and *F*), indicating that DAD-mediated autoinhibitory interactions do not require GBD.

**Figure 7:**
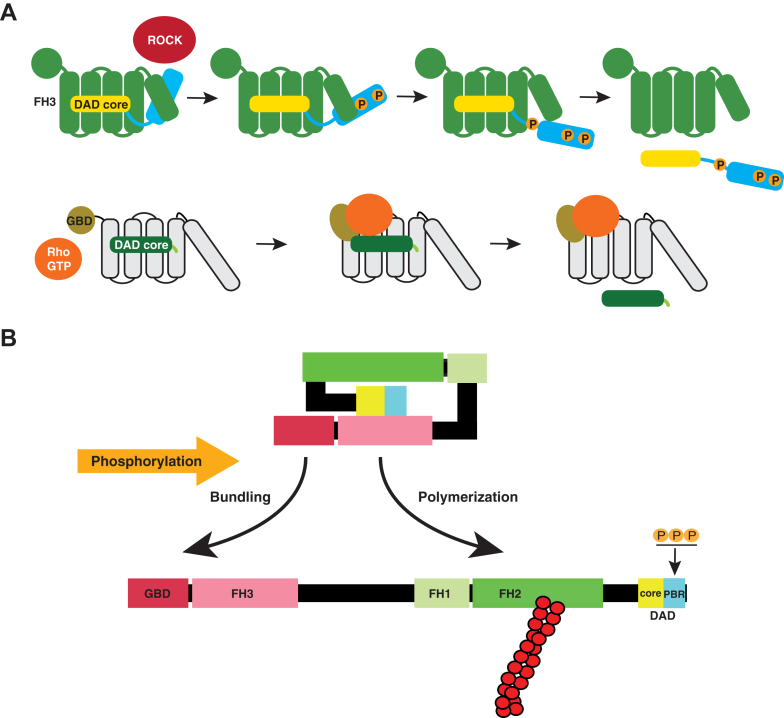
**A proposed model for the activation process of human FHOD1.***A,* schematic model of the stepwise process of DAD release from the FH3 domain of FHOD1 by ROCK-mediated phosphorylation (*upper part*) and that of DIAPH1 by Rho binding (*lower part*), as described in the text. *B,* schematic representation of the integrated model of domain organization and molecular regulation of human FHOD1. The autoinhibitory interaction between FH3 and the bipartite DAD is relieved by ROCK-induced phosphorylation within the extended polybasic region, leading to FH2-mediated actin polymerization and GBD-mediated colocalization of FHOD1 with stress fibers, resulting in the formation of bundled stress fibers. The surface of the complex is shown. DAD, diaphanous autoregulatory domain; FHOD, formin homology domain–containing protein; GBD, GTPase-binding domain; ROCK, Rho-associated kinase.

It is noteworthy that ΔN93-R1128D, ΔN93-R1135D, and ΔN93-R1139E exhibited thinner, less bundled stress fibers than FL-3xD (Fig. 6, *E* and *G*) and FL-R1128D, FL-R1135D, and FL-R1139E (Fig. 2, *D* and *F*). In addition, GBD-containing full-length active forms, FL-3×D, FL-R1128D, FL-R1135D, and FL-R1139E, were found to be colocalized with bundled thick stress fibers, whereas GBD-lacking active forms, ΔN93-R1128D, ΔN93-R1135D, and ΔN93-R1139E, were less colocalized with stress fibers (Figs. 2*D* and 6*E*). These findings suggest that GBD is responsible for both FHOD1 localization to stress fibers and the thickening of stress fibers. It is conceivable that GBD may mediate the localization of FHOD1 to stress fibers and bundling of stress fibers *via* an unverified mechanism (Fig. 7*B*). Thus, it seems likely that the GBD is not involved in the regulation of autoinhibition; rather, its role appears to be in the regulation of the localization and function of FHOD1.

## Discussion

In the present study, we showed that electrostatic interactions mediated by the extended polybasic region, which is unique to the FHOD subfamily, are critical for the regulation of the autoinhibitory interaction of FHOD1. The extended region expands the electrostatically interacting surfaces to stabilize the structure of the autoinhibitory complex. Concurrently, the extended region appeared to function as a switch for activation upon phosphorylation. The distinctive nature of the autoinhibitory interaction of FHOD1 was successfully elucidated by integrating protein modeling using AlphaFold3 with biochemical analyses. Thus, the AlphaFold3-guided structure prediction is anticipated to refine the interpretation of the experimental protein–protein interaction analysis.

The activity of the catalytic unit FH1–FH2 of mammalian formin DIAPH1 is normally inhibited by the autoinhibitory interaction between the N- and C-terminal regions (6). Autoinhibition of DIAPH1 is mediated by the core motif MDXLLXL and the basic residues RRKR (23). The importance of the RRKR in the autoinhibition of DIAPH is demonstrated by the observation that truncation of the RRKR in human DIAPH1 results in a constitutively active form, consequently leading to hearing loss (24). However, despite their importance, RRKR residues were not visible in the crystal structure of the complex (9). A recent NMR study revealed only a transient interaction between the basic motif of DAD and DID, indicating flexibility of the RRKR motif (25). The flexible and transient interaction of the RRKR motif appears to contribute to the formation of a productive encounter complex, which facilitates an autoinhibited state by increasing the probability of encountering DAD with DID in the context of full-length DIAPH1.

FHOD1 also harbors a DAD core motif and the following basic residues, both of which are required for autoinhibition (20). Notably, the basic residues in FHOD are extended C-terminally compared with DIAPH, and the extended region expands the electrostatic interacting surfaces to stabilize the structure of the autoinhibitory complex. Furthermore, the current predicted model showed that a side chain of R1139 within the extended region penetrates, like a hook, into a spherical hole in the negatively charged cavity of FH3 (Fig. 2*A*). This terminal hook-like structure significantly enhanced the binding energy between FH3 and DAD. Thus, the extended polybasic region in FHOD1 plays a critical role in enhancing this interaction. At the same time, this unique extended region contains conserved phosphorylatable residues S1131, S1137, and T1141. The predicted model with phosphorylation of these residues demonstrated destabilization of the autoinhibitory interaction, which is in good agreement with our previous experimental data (14). Thus, the extended region appears to play an ambivalent role in the regulation of FHOD1, both in stabilization and release of autoinhibition.

In the case of DIAPH1, Rho appears to only partially relieve the DAD-mediated autoinhibition of actin assembly (29), indicating the involvement of additional factors or mechanisms in the activation process, which appears to allow gradual activation *in vivo* to achieve cellular processes requiring prolonged and controlled cytoskeletal remodeling (30). Consistently, FHOD1 activation may also be achieved gradually. As previously demonstrated, ROCK could phosphorylate S1137 and T1141 of DAD in the presence of the N terminus of FHOD1, whereas phosphorylation of S1131 was prevented by the presence of the N terminus of FHOD1 (14). The current predicted structural model can provide a satisfactory explanation of the experimental results. As demonstrated in Fig. S9, T1141 was predominantly exposed on the surface of the complex. The proximity of S1137 to T1141 may facilitate the concurrent phosphorylation of S1137. The resultant double phosphorylation of S1137 and T1141 may serve to disengage the interaction, thus facilitating the subsequent phosphorylation of S1131, which ultimately culminates in the dissolution of autoinhibition (Fig. 7*A*). Thus, it can be postulated that sequential phosphorylation within the polybasic region plays a pivotal role in gradual activation.

It has recently been reported that the GBD of FHOD1, inconsistent with previous expectations, does not bind to small GTPase Rac1 (28). Instead, the GBD binds to spectrin repeats of nesprin, a nuclear membrane–binding protein, and plays a role in nuclear positioning. In contrast, our present results support the role of GBD in stress fiber colocalization and actin bundling, which was previously predicted (15). As shown in Figure 6, active FHOD1 lacking GBD was not localized to stress fibers, and the stress fibers formed were thinner than those induced by active FHOD1 harboring GBD. Although the bundling activity of FHOD1 usually seems to be inhibited by the intramolecular interaction mediated by DID and DAD, the release of the autoinhibition appears to induce not only FH2-mediated polymerization but also GBD-mediated bundling (Fig. 7*B*).

A series of N-terminally truncated constructs of FHOD1 (Fig. 6*A*) were designed based on transcript variants with alternative transcription start sites present in databases. Although these variants were only computationally predicted and not experimentally confirmed, it is possible that these variants lacking GBD play a role in a physiological context. It has been demonstrated that FHOD3, a homolog of FHOD1 associated with hypertrophic cardiomyopathy, has several splicing variants that are implicated in the pathogenesis of cardiomyopathy (31, 32). The existence of FHOD1 splicing variants and their physiological significance should be elucidated in future studies.

Although the formin family comprises 15 members, the regulatory mechanisms of formins have been extrapolated from the findings obtained from the prototypic formin DIAPH1. However, each member seems to have a unique regulatory mechanism that appears to be responsible for a variety of physiological and pathological functions. The present study demonstrates the structural basis underlying the unique autoinhibition and phosphorylation-dependent activation processes of FHOD1. The utilization of information derived from prediction models is expected to enhance our understanding of the divergent regulatory mechanisms of formins under various pathophysiological conditions.

## Experimental procedures

### Plasmid construction

The human complementary DNA (cDNA) fragments encoding FHOD1-FL (amino acids 1–1164), FHOD1-N1 (1–569), and FHOD1-DAD (1081–1145) were prepared as described previously (14). The cDNA for FHOD1-N2 (1–441), FHOD1-N3 (1–360), FHOD1-ΔN93 (94–1164), FHOD1-ΔN187 (188–1164), and FHOD1-ΔN305 (306–1164) were amplified from the human FHOD1 cDNA by PCR using specific primers. Mutations leading to the indicated amino acid substitutions were introduced by PCR-mediated site-directed mutagenesis. The DNA fragments were ligated to pGEX-6P (GE Healthcare Bio-Sciences) or pProEX-HTb (Invitrogen) for bacterial expression as a protein fused to GST or a His-tagged protein, respectively. For expression as a protein fused to GFP in mammalian cells, the fragments were ligated to pEGFP-C1 (Clontech). All the constructs were sequenced for confirmation of their identities.

### Protein expression and purification

Protein expression and purification were performed as previously described with minor modifications (14). Briefly, *Escherichia coli* strain BL21 transformed with a plasmid DNA encoding GST-fused or His-tagged protein was cultured in 2x YT medium at 37 °C until the absorbance at 600 nm reached 0.5 to 0.8. Protein expression was induced by adding 0.5 mM IPTG and incubating for 2 h at 37 °C. The cells were then collected by centrifugation at 5000*g* for 15 min. The cell pellet was resuspended in PBS and stored at −80 °C. The resuspended cells were sonicated, and the lysate was clarified by centrifugation at 15,000*g* for 30 min at 4 °C. The supernatant was then applied to glutathione-Sepharose-4B (GE Healthcare) or HIS-Select Nickel Affinity gel (Sigma–Aldrich) for protein purification.

### Pull-down binding assay

Pull-down binding assays were performed as previously described with minor modifications (33). Briefly, 20 μg of GST fusion protein and 20 μg of His-tagged protein were mixed in 500 μl PBS (137 mM NaCl, 2.68 mM KCl, 8.1 mM Na_2_HPO_4_, and 1.47 mM KH_2_PO_4_, pH 7.4) containing 0.1% Triton X-100 (final concentrations of GST-DAD and His-FHOD1-N3 were 1.25 μM and 1.0 μM, respectively). A slurry of glutathione-Sepharose 4B was added to the mixture and incubated for 1 h at 4 °C. After washing three times with PBS containing 0.1% Triton X-100, proteins were eluted from glutathione-Sepharose 4B with 10 mM glutathione. The eluates were subjected to SDS-PAGE and stained with Coomassie Brilliant Blue (CBB). Densitometric analysis of the CBB staining was performed using Fiji (34).

In the case of quantitative analysis (Fig. S6*F*), His-tagged FHOD1-N3 (1–360) was incubated with the indicated concentrations of GST-fused FHOD1 DAD (1081–1145) with MagneGST glutathione particles in 100 μl PBS containing 0.05% Triton X-100. Bound proteins were collected with glutathione particles without washing, subjected to SDS-PAGE, and analyzed by CBB staining followed by densitometric measurement using the image analyzer LAS-4000 (Fuji Photo Film).

### Cells and immunofluorescence staining

Cell culture and immunofluorescence staining were performed as previously described, with minor modifications (14). Briefly, HeLa cells were cultured in Dulbecco’s modified Eagle’s medium (DMEM) supplemented with 10% fetal bovine serum (FBS). Prior to transfection, the medium was replaced with DMEM supplemented with 2% FBS for 1 to 2 h. The cells were then transfected with plasmids using Transporter 5 (Polysciences) and cultured for 4 h. After replacing the medium with DMEM supplemented with 10% FBS, the cells were cultured for another 14 to 15 h. After washing three times with PBS, the cells were fixed for 15 to 20 min in 3.7% formaldehyde in PBS. The cells were permeabilized by washing five times with 0.1% Triton X-100 in PBS. F-actin staining was performed using Alexa Fluor 568 Phalloidin (Invitrogen). Nuclear staining was performed using Hoechst 33342 (Fujifilm Wako Chemicals). Images were visualized and captured using an IXplore SpinSR10 confocal microscope (Olympus) or an FLUOVIEW FV4000 confocal microscope (Olympus).

### Quantitative analysis of stress fiber formation

Quantification of stress fiber formation was conducted in accordance with the following criteria. Extensive stress fiber formation was defined as the percentage of cells showing stress fiber formation that exceeds 50% of the cell area. Thick stress fiber formation was defined as the percentage of cells showing five or more thick, bundled stress fibers with a diameter of 1.0 μm or greater. For each mutant, the percentages were calculated from a total of 20 to 30 cells in a single transfection experiment, and the mean ± SD was obtained from three independent transfection experiments.

### Model building

The structure of the autoinhibitory complex between the N-terminal region and DAD of human FHOD1 was predicted using AlphaFold3 (https://alphafoldserver.com/) (19). The prediction process was repeated three times with the default setting, resulting in 15 structural predictions (five structure models per seed). The model is available in ModelArchive at https://modelarchive.org/doi/10.5452/ma-oqu94/. Models were visualized using UCSF ChimeraX (https://www.cgl.ucsf.edu/chimerax/). The top-ranked model, as determined by the AlphaFold3 ranking score, was further analyzed using the web analysis tool PDBsum (22).

### Analysis of amino acid interactions and interaction energies

The energetic contributions of individual amino acid residues in the predicted protein structure were analyzed using the INTAA web server, version 2.0 (https://bioinfo.uochb.cas.cz/INTAA/) (26). To ascertain the contribution of individual amino acids within the predicted model of the complex structure of the N terminus and DAD of FHOD1 by AlphaFold3, the interaction energies between the side chains of individual residues were calculated. The sum of interaction energies between residues in the N terminus and DAD was calculated from the net interaction energies of all residues of each peptide chain. For each analysis, 15 predicted models from three independent runs of prediction were used.

The binding energy between the N terminus and DAD was also predicted based on the predicted structure using the PROtein binDIng enerGY prediction (PRODIGY) web server (https://rascar.science.uu.nl/prodigy/) (27).

### Analysis of sequence conservation

The sequence conservation of DAD among DIAPH and FHOD subfamilies was analyzed using the ConSurf webserver (https://consurf.tau.ac.il/consurf_index.php) (35). Briefly, we entered the indicated structure into the ConSurf webserver to conduct a homolog search, generate a multiple sequence alignment, and calculate evolutionary conservation scores. The resulting conservation scores were then visualized using WebLogo (https://weblogo.berkeley.edu/logo.cgi) (36).

### Statistical analysis

Data are presented as mean ± SD. Statistical analyses were performed using GraphPad Prism, version 10 (GraphPad Software). To compare the mean values of two groups, an unpaired *t* test was used. Differences between the mean values of three or more groups were analyzed by one-way analysis of variance followed by post hoc Dunnett's test. A *p* value of <0.05 was considered to be statistically significant.

## Data availability

All data described in this study are available either within the article or the supporting information.

## Supporting information

This article contains supporting information (22, 26, 27, 36)`

## Conflict of interest

The authors declare that they have no conflicts of interest with the contents of this article.

## Supporting information

Supplemental Tables legend

Supplementary Table 1 Multiple sequence alignment of DAD sequences of the DIAPH subfamily. 119 DAD sequences of the DIAPH subfamily were used to generate the graphical representation of multiple sequence alignment shown in Fig. S4*E*.

Supplementary Table 2 Multiple sequence alignment of DAD sequences of the FHOD subfamily. 116 DAD sequences of the FHOD subfamily were used to generate the graphical representation of multiple sequence alignment shown in Fig. S5*A*.
