# Supplementary material for: Structural basis underlying the autoinhibition of the formin FHOD1 and its phosphorylation-dependent activation
Source: J Biol Chem. 2025 Dec 23;302(2):111109. doi: 10.1016/j.jbc.2025.111109 (PMC12858348; doi:10.1016/j.jbc.2025.111109)
Supplement: Supplementary Figure 1 [file mmc1.pdf]

Supplementary Fig 1. Syaban et al

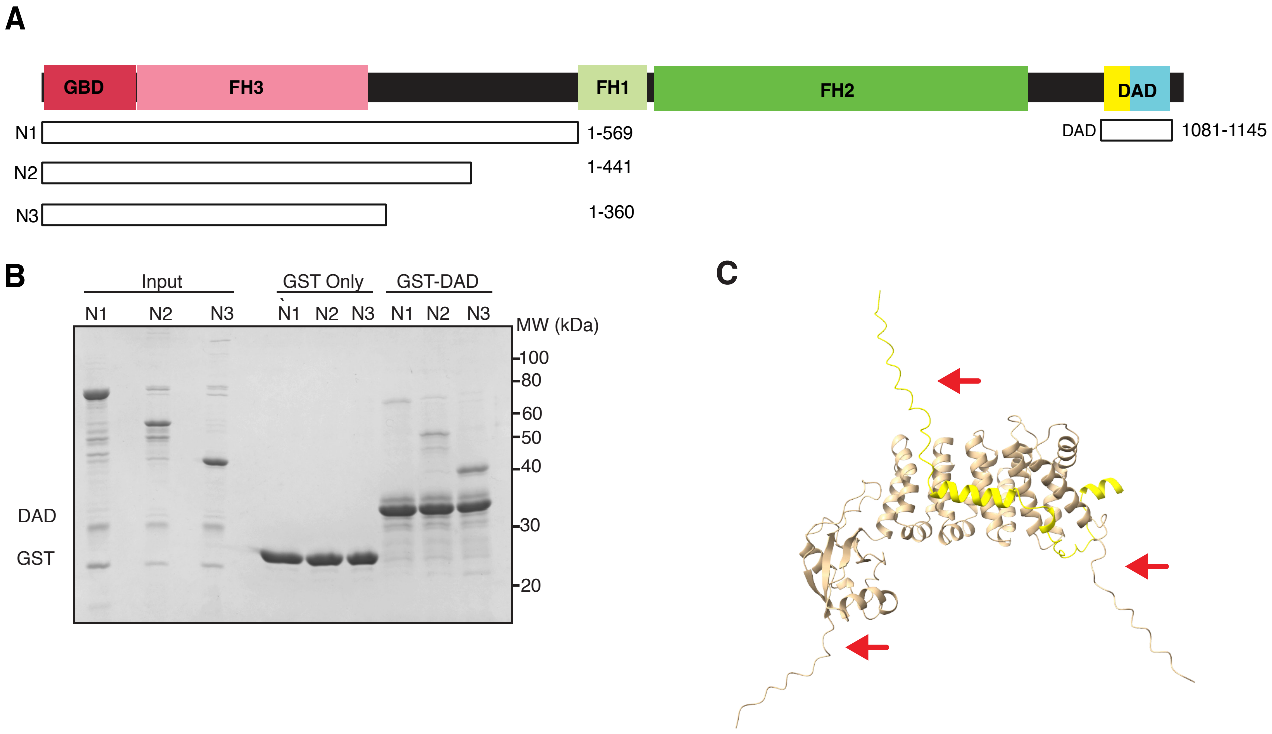

Supplementary Figure 1. Minimizing the regions sufficient for FHOD1 autoinhibitory interaction. A, schematic representation of the domain architecture of human FHOD1. The fragments used for pull-down assay shown in (B) are indicated by white boxes. B, GST pull-down assay using a truncated form of the N-terminal region of FHOD1. The indicated truncated form of His-tagged FHOD1-N was incubated with GST-fused FHOD1-DAD (1081–1145). Proteins were pulled down with glutathione–Sepharose-4B and the precipitants were subjected to SDS–PAGE followed by CBB staining. C, predicted structure of the autoinhibitory complex consisting of FHOD1-N3 (1–360) (beige) and DAD (1081–1145) (yellow) of FHOD1. These fragments were used in the pull-down assay in the present study. Unstructured regions are indicated by red arrows.
