# Supplementary material for: Structural basis underlying the autoinhibition of the formin FHOD1 and its phosphorylation-dependent activation
Source: J Biol Chem. 2025 Dec 23;302(2):111109. doi: 10.1016/j.jbc.2025.111109 (PMC12858348; doi:10.1016/j.jbc.2025.111109)
Supplement: Supplementary Figure 2 [file mmc2.pdf]

Supplementary Fig 2. Syaban et al

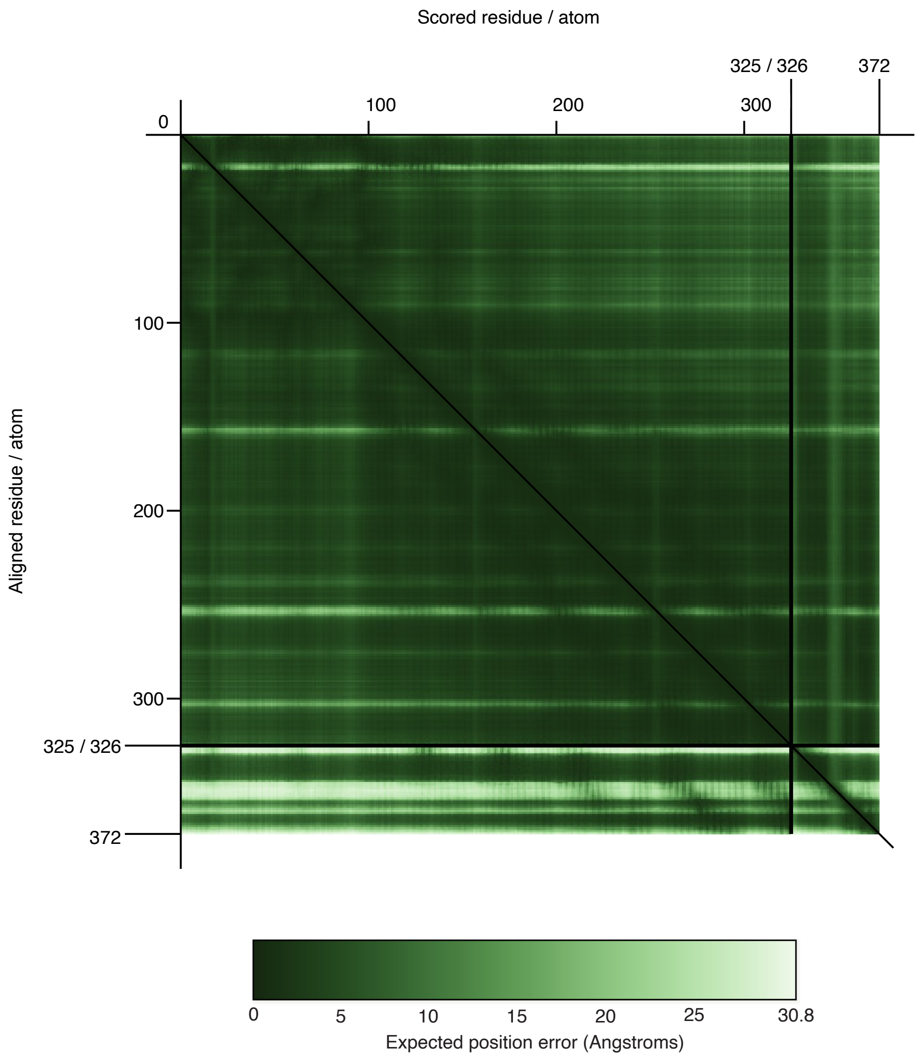

Supplementary Figure 2. The predicted aligned error (PAE) plot of the predicted complex structure of the N-terminal region (15–339) and DAD (1099–1145) of FHOD1. The residues 1–325 and 326–372 in the plot correspond to the N-terminal region (15–339) and DAD (1099–1145), respectively.
