# Supplementary material for: Structural basis underlying the autoinhibition of the formin FHOD1 and its phosphorylation-dependent activation
Source: J Biol Chem. 2025 Dec 23;302(2):111109. doi: 10.1016/j.jbc.2025.111109 (PMC12858348; doi:10.1016/j.jbc.2025.111109)
Supplement: Supplementary Figure 3 [file mmc3.pdf]

A

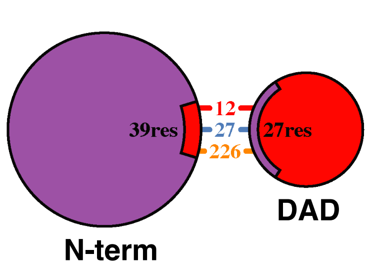

	No. of interface residues	Interface area (Å^2)	No. of salt bridges	No. of disulphide bonds	No. of hydrogen bonds	No. of non-bonded contacts
N-term	39	1534	12	-	27	226
DAD	27	1782				

B

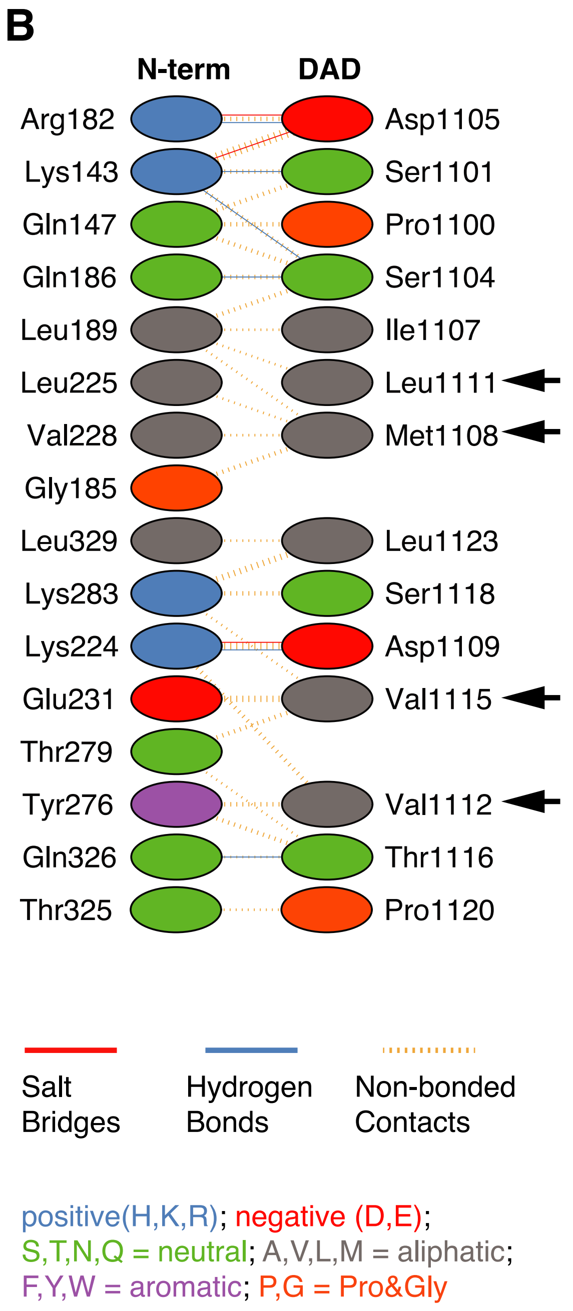

C

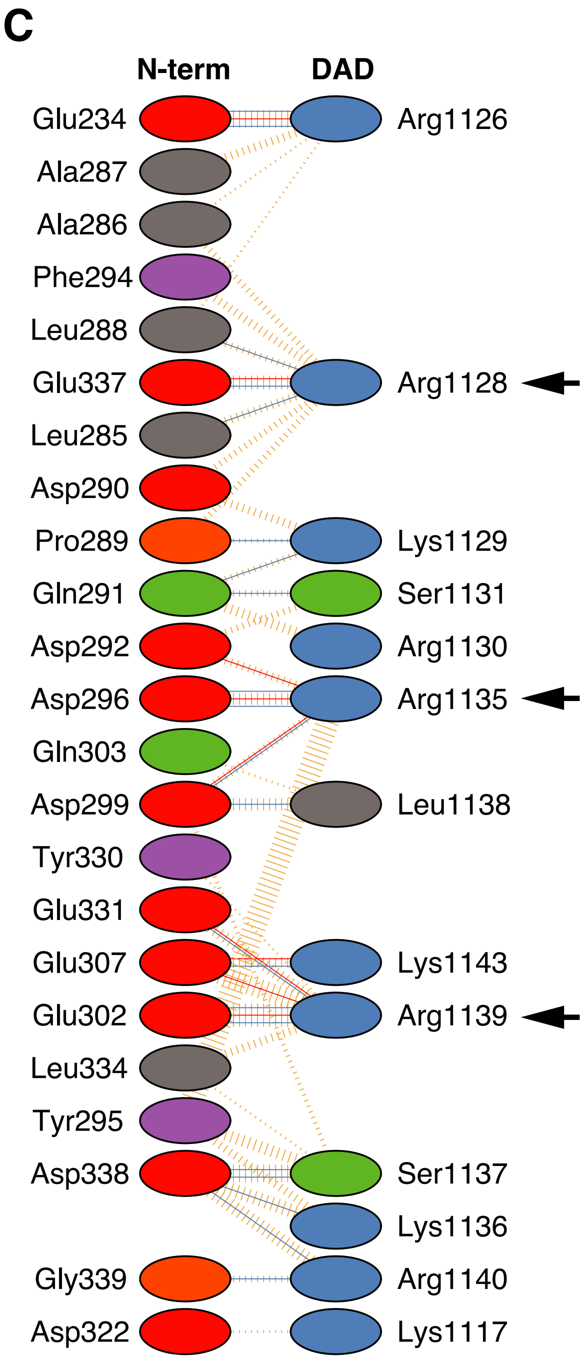

Supplementary Figure 3. The structural analysis for the autoinhibitory complex between the N-terminus and DAD of FHOD1. A, summary of interactions between the N-terminal region and DAD of FHOD1 including the number and types of interactions and the interface area involved in binding, which is analyzed by the web analysis tool PDBsum (22). B and C, residue interactions across interface of the complex. Interacting residues across the interface of FH3 with the DAD core motif (B) or with the polybasic region (C) are connected by colored lines representing different types of interactions. Interacting residues are color-coded by residue type. The residues for which substitutions were introduced in this study are indicated by arrows.
