# Supplementary material for: Structural basis underlying the autoinhibition of the formin FHOD1 and its phosphorylation-dependent activation
Source: J Biol Chem. 2025 Dec 23;302(2):111109. doi: 10.1016/j.jbc.2025.111109 (PMC12858348; doi:10.1016/j.jbc.2025.111109)
Supplement: Supplementary Figure 4 [file mmc4.pdf]

Supplementary Fig 4. Fahmi et al

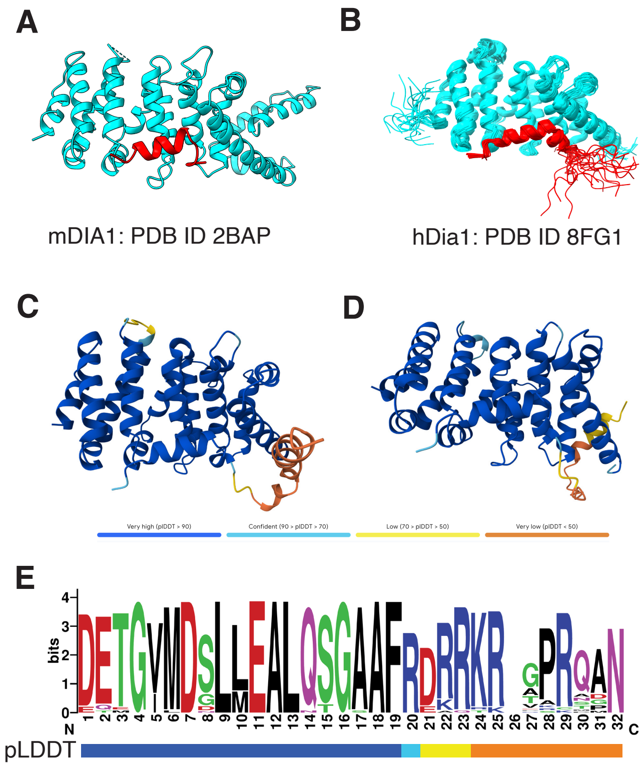


**Supplementary Figure 4. The autoinhibitory interaction between DAD and FH3 domain of DIAPH1.** A and B, the crystal structure of the complex between FH3 and DAD of mouse DIAPH1 (A) and the solution structure of human DIAPH1 (B). C and D, the AlphaFold3-predicted structure of the complex between FH3 and DAD of mouse DIAPH1 (C) and that of human DIAPH1 (D) colored by pLDDT score, as shown in Figure 1D. E, the sequence conservation among 119 DAD sequences of DIAPH subfamily (Table S1) was projected on the sequence by WebLogo (35). The pLDDT scores for each residue shown in C and D are indicated by color below the respective sequence.
