# Supplementary material for: Structural basis underlying the autoinhibition of the formin FHOD1 and its phosphorylation-dependent activation
Source: J Biol Chem. 2025 Dec 23;302(2):111109. doi: 10.1016/j.jbc.2025.111109 (PMC12858348; doi:10.1016/j.jbc.2025.111109)
Supplement: Supplementary Figure 5 [file mmc5.pdf]

Supplementary Fig 5. Syaban et al

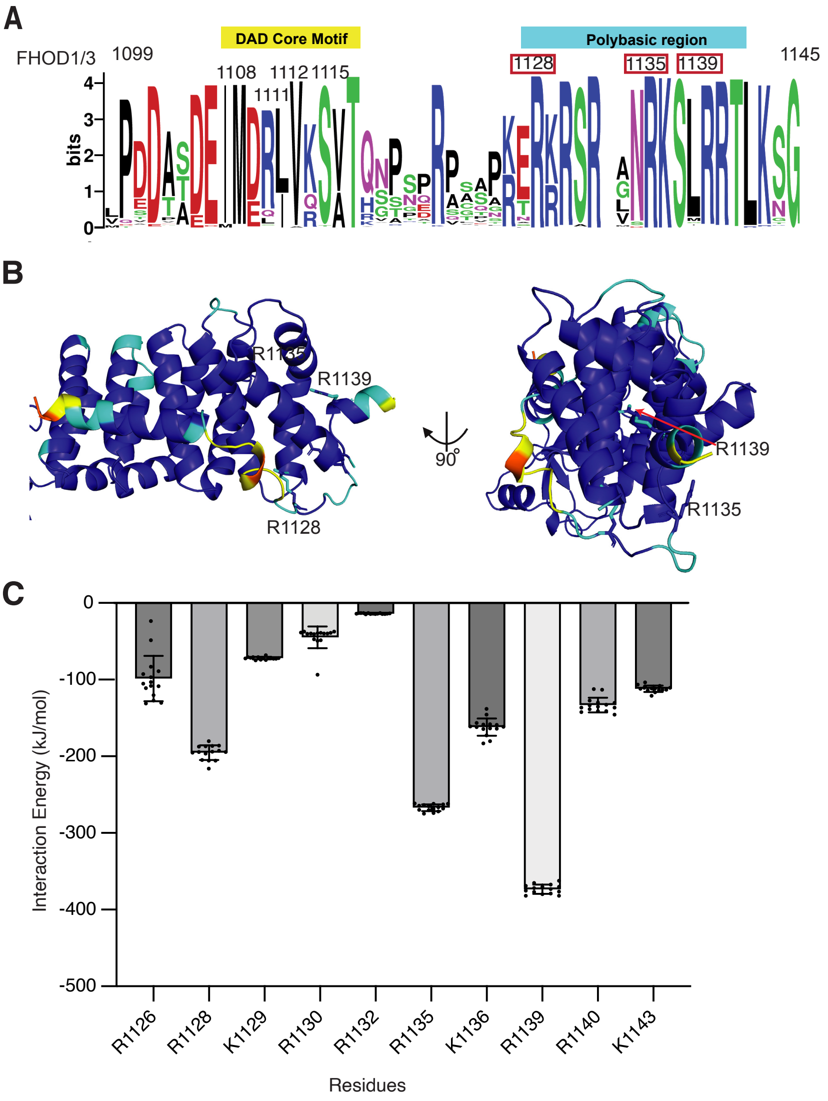

Supplementary Figure 5. The basic residues in the polybasic region of FHOD1-DAD. A, the sequence conservation among 116 DAD sequences of FHOD subfamily (Table S2) was projected on the sequence by WebLogo (35). B, positions and pLDDT values of the basic residues R1128, R1135, and R1139 in the predicted model. The color-coding of pLDDT score is shown in Figure 1D. C, residue-specific interaction energy in the complex of the N-terminus and DAD of FHOD1. The interaction energies of the indicated basic residues in the polybasic region are calculated by the INTAA web server (26) using 15 predicted structure models. Data are presented as dot plots and mean ± S.D.
