# Supplementary material for: Structural basis underlying the autoinhibition of the formin FHOD1 and its phosphorylation-dependent activation
Source: J Biol Chem. 2025 Dec 23;302(2):111109. doi: 10.1016/j.jbc.2025.111109 (PMC12858348; doi:10.1016/j.jbc.2025.111109)
Supplement: Supplementary Figure 6 [file mmc6.pdf]

Supplementary Fig 6. Syaban et al

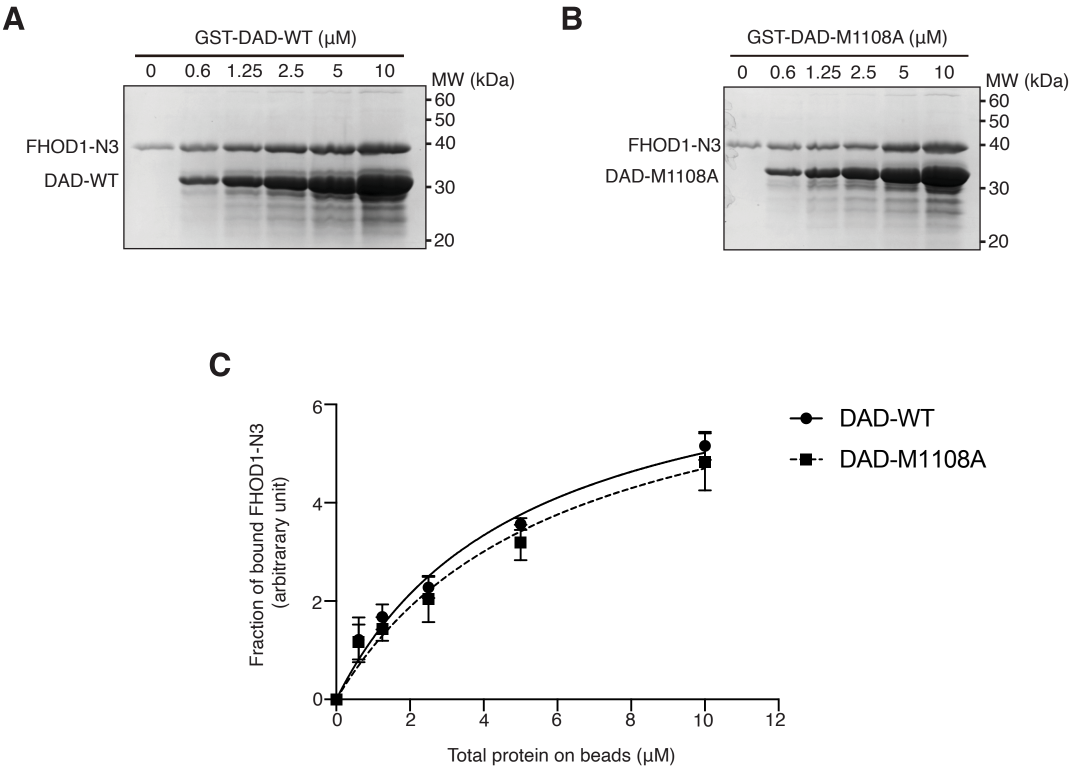

Supplementary Figure 6. Quantitative analysis of binding of FHOD1-DAD with the N-terminal region. *A* and *B*, representative pull-down assay for quantification of binding of FHOD1-DAD with the N-terminal region. His-tagged FHOD1-N3 (1–360) was incubated with the indicated concentrations of GST-fused FHOD1 DAD (1081–1145) WT (A) or M1108A (B) with MagneGST glutathione particles. Bound proteins were collected with glutathione particles without washing, subjected to SDS-PAGE, and analyzed by CBB staining. *C*, quantitative analysis for binding of FHOD1-N3 to FHOD1-DAD. Fractions of specifically bound FHOD1-N were determined as fraction bound to GST–DAD immobilized to MagneGST glutathione particles minus fraction bound to MagneGST glutathione particles in A and B. The estimated dissociation constant (K_d) values from three independent experments for WT and M1108A were 5.1 ± 0.9 μM and 6.0 ± 1.6 μM, respectively.
