# Supplementary material for: Structural basis underlying the autoinhibition of the formin FHOD1 and its phosphorylation-dependent activation
Source: J Biol Chem. 2025 Dec 23;302(2):111109. doi: 10.1016/j.jbc.2025.111109 (PMC12858348; doi:10.1016/j.jbc.2025.111109)
Supplement: Supplementary Figure 7 [file mmc7.pdf]

Supplementary Figure 7. Syaban et al

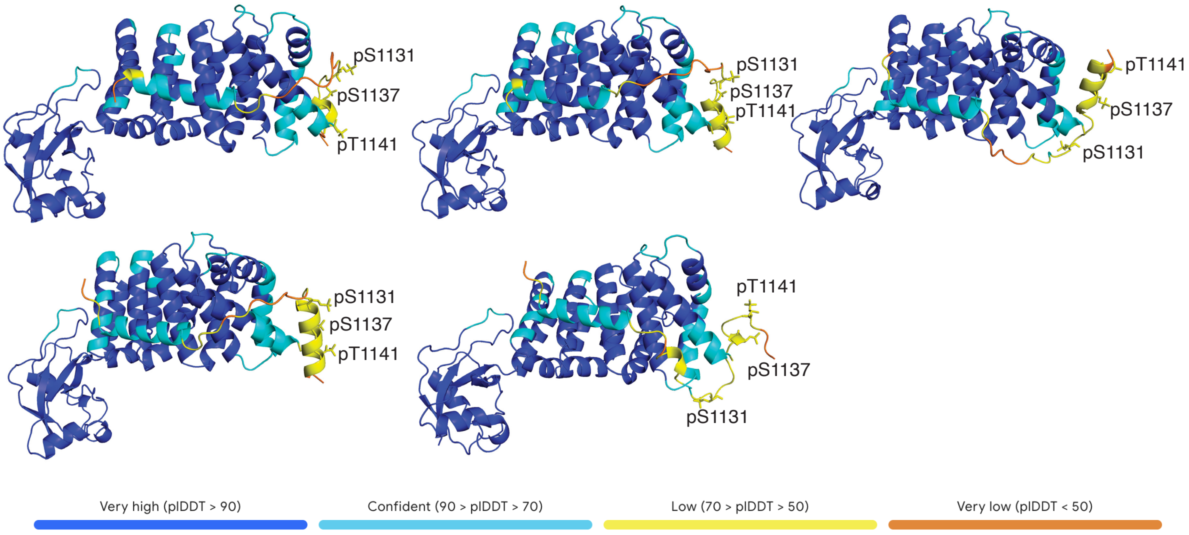

Supplementary Figure 7. Predicted structure models of the phosphorylated autoinhibitory complex. Top 5 predicted models of the complex of DAD phosphorylated at S1131, S1137, and T1141.
