# Supplementary material for: Structural basis underlying the autoinhibition of the formin FHOD1 and its phosphorylation-dependent activation
Source: J Biol Chem. 2025 Dec 23;302(2):111109. doi: 10.1016/j.jbc.2025.111109 (PMC12858348; doi:10.1016/j.jbc.2025.111109)
Supplement: Supplementary Figure 8 [file mmc8.pdf]

Supplementary Figure 8. Syaban et al

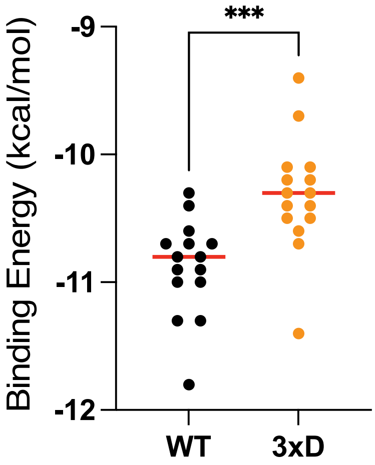

Supplementary Figure 8. The predicted binding energy between the N-terminus and DAD. The binding energy between the N-terminus (15–339) and DAD (1099–1145) was predicted using the PRODIGY web server (https://rascar.science.uu.nl/prodigy/) (27) with 15 AlphaFold3-predicted models. Since this web server accepts only protein structures composed of standard non-phosphorylated amino acids as input, the triple substituted form of aspartate for S1131, S1137, and T1131 (3×D) was used as a mimic of the phosphorylated form. Data are presented as dot plots and mean ± S.D. ***, $p < 0.001$.
