# Supplementary material for: Structural basis underlying the autoinhibition of the formin FHOD1 and its phosphorylation-dependent activation
Source: J Biol Chem. 2025 Dec 23;302(2):111109. doi: 10.1016/j.jbc.2025.111109 (PMC12858348; doi:10.1016/j.jbc.2025.111109)
Supplement: Supplementary Figure 9 [file mmc9.pdf]

Supplementary Figure 9. Fahmi et al

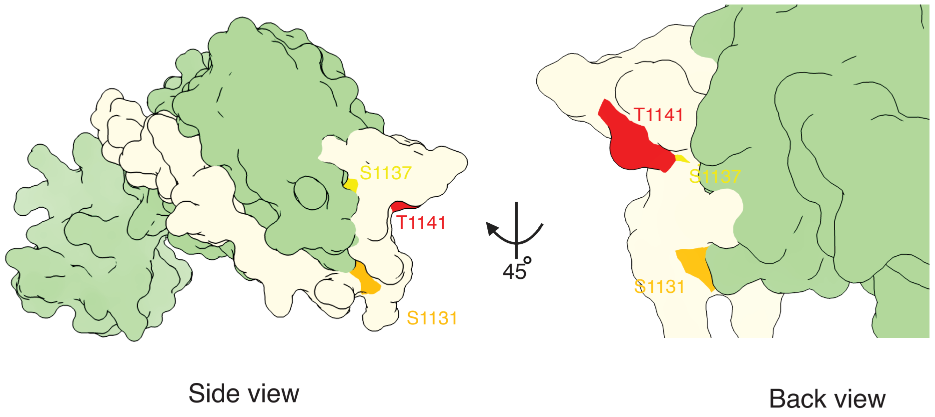

Supplementary Figure 9. Surface structure of the predicted model of the complex of the N-terminus and DAD of FHOD1. The extent of exposure of phosphorylatable residues S1131, S1137, and T1141 to the surface of the complex are shown.
