# Supplementary material for: Structural basis underlying the autoinhibition of the formin FHOD1 and its phosphorylation-dependent activation
Source: J Biol Chem. 2025 Dec 23;302(2):111109. doi: 10.1016/j.jbc.2025.111109 (PMC12858348; doi:10.1016/j.jbc.2025.111109)
Supplement: Supplemental Tables legend [file mmc10.docx]

**Supplementary Table 1. Multiple sequence alignment of DAD sequences of the DIAPH subfamily.** 119 DAD sequences of the DIAPH subfamily were used to generate the graphical representation of multiple sequence alignment shown in Figure S4E.

**Supplementary Table 2. Multiple sequence alignment of DAD sequences of the FHOD subfamily.** 116 DAD sequences of the FHOD subfamily were used to generate the graphical representation of multiple sequence alignment shown in Figure S5A.
